# Tying the Subclavian Artery within the Scaleni Muscles of the Left Side for Aneurism

**Published:** 1845-12

**Authors:** 


*Tying the Sub-clavian Artery within the Scaleni muscles of the left side for Aneurism.*—Dr. Rodgers had a good subject, and performed the operation in the most skilful manner, assisted by Dr. Cheesman and others. The patient had every requisite attention paid him by the house surgeon, besides the daily attendance of Dr. R. Most of the Surgeons of the Institution were in favor of the operation, although some entertained doubts as to the result. The following is a brief history of the case:

Michæl Larman, æt. 42, (born in Germany,) laborer—entered the New York Hospital, Sept. 13th, 1845. Admitted with aneurism of the left sub-clavian artery. About four months previously, he felt great pain about the left shoulder and arm, and slight swelling. Four or five weeks previous to the operation, he was attacked with pain and soreness, when carrying a basket of peaches on his shoulder, and, for the first time, he discovered a small tumor just above the collar bone; and when admitted, it was about the size of a goose's egg, presenting a strong pulsatory character, its outer margin reaching by the sterno-cleido-mastoid muscle, and extending to the outer third of the clavicle. On every side, pulsation could be distinctly felt. The cutaneous veins over the tumor were much enlarged, and the arm, forearm, and hand, were considerably swollen. He complained of severe pain in the axilla, extending to the ends of the fingers, and was unable to sleep on account of the pressure and pulsation of the tumor on the nerves. The patient was bled ℥x., and put upon ℞. Tr. Digit. gtt. xxx., ter in die. The treatment was continued with occasional anodynes and purgatives, and V. S. twice more at intervals, up to the day of the operation.

The operation was performed in the following manner; an L incision was made from the middle of the thyroid cartilage, a little to the left of the median line down to the sternum, and along the clavicle to its outer third; the veins ramifying over the integuments, bled copiously and required the ligature. The flap was dissected up, and the sterno-cleido-mastoid muscle was separated from its attachment; there were one or two small arteries in the substance of the muscle, that required the ligature.

The deep fascia of the neck was divided by means of the director and bistoury, and the rest of the structure was separated by means of the handle of the scalpel and fingers. The artery was tied just on the inside of the scalena anticus. The pulsation ceased in the tumor, directly after tightening the ligature.

The patient seemed to be doing remarkably well until the 26th of the month, and twelfth day of the operation, when secondary hemorrhage commenced; every means was used to arrest it, but without avail; he died at five o'clock, P. M., Oct. 28th, and the fourteenth day of the operation.

The hemorrhage was from the sub-clavian, and followed the track of the ligature. We have not been able to obtain the particulars of the autopsy yet, but we understand Dr. Rodgers feels more assured of the propriety of the operation since examining the parts after death.—*N. Y. Med. and Surg. Reporter.*

